# A novel *microbe-based* treatment that attenuates the inflammatory profile in a mouse model of allergic airway disease

**DOI:** 10.1038/srep35338

**Published:** 2016-10-13

**Authors:** Mark Bazett, Agnieszka Biala, Ryan D. Huff, Momir Bosiljcic, Hal Gunn, Shirin Kalyan, Jeremy A. Hirota

**Affiliations:** 1 Qu Biologics Inc., Vancouver, BC, V5T 4T5, Canada; 2 University of British Columbia, Department of Medicine, Division of Respiratory Medicine, Vancouver, BC, V6H 3Z6, Canada; 3 University of British Columbia, Department of Medicine, Division of Endocrinology, CeMCOR, Vancouver, BC, V5Z 1M9.

## Abstract

There is an unmet need for effective new and innovative treatments for asthma. It is becoming increasingly evident that bacterial stimulation can have beneficial effects at attenuating allergic airway disease through immune modulation. Our aim was to test the ability of a novel inactivated microbe-derived therapeutic based on *Klebsiella* (KB) in a model of allergic airway disease in mice. BALB/c mice were exposed intranasally to house dust mite (HDM) for two weeks. Mice were treated prophylactically via subcutaneous route with either KB or placebo for one week prior to HDM exposure and throughout the two week exposure period. 24 hours after the last exposure, lungs were analysed for inflammatory cell infiltrate, gene expression, cytokine levels, goblet cell metaplasia, and serum was analysed for allergen-specific serum IgE levels. HDM exposed mice developed goblet cell hyperplasia, elevated allergen-specific serum IgE, airway eosinophilia, and a concomitant increase in T_H_2 cytokines including IL-4, IL-13 and IL-5. Treatment with KB attenuated HDM-mediated airway eosinophilia, total bronchoalveolar lavage (BAL) cell numbers, BAL T_H_2 cytokine production, and goblet cell metaplasia. Our prophylactic intervention study illustrates the potential of subcutaneous treatment with bacterial derived biologics as a promising approach for allergic airway disease treatment.

Asthma is a chronic inflammatory lung disease characterized by reversible airflow obstruction and airway hyperresponsiveness in response to environmental stimuli^1^. Airway inflammation includes an alteration in the profile and magnitude of cytokines that are locally produced, and the associated recruitment and activation of immune cells^2^. It is estimated that up to 300 million people^3^,^4^ suffer from asthma worldwide with the standard of care involving stepwise therapies designed to control asthma symptoms. This includes β-agonists, inhaled corticosteroids, combination therapies, and biologics^5^. Despite these therapeutic approaches, a subset of patients are not adequately controlled with current treatment options, and no therapeutic approach exists to reverse established asthma.

Asthma has multiple endotypes that are defined by cellular and immune mediator profiles^2^,^6^,^7^. A large proportion of asthmatics demonstrate a T_H_2-cytokine skewed inflammatory profile^2^. The T_H_2-skewed asthmatic population frequently presents with an allergic phenotype characterized by increased allergen-specific serum immunoglobulin E (IgE) antibodies, lung eosinophilia, and increased bronchoalveolar lavage (BAL) T_H_2 cytokine levels including IL-4, IL-5 and IL-13^1^,^6^,^8^. This T_H_2 signature is currently being targeted with new biologics, including antibodies against IL-4Rα^9^, IL-5^10^, and IL-13^11^, which have provided clinical benefit to select patient populations. The clinical efficacy of these biologics gives evidence that strategies that are able to alter the T_H_2-skewing of the immune response in asthma would be of significant benefit.

The hygiene hypothesis, and adapted variations, has been proposed to explain the increase in rates of asthma in developed countries^12^,^13^. This hypothesis broadly states that there is a protective influence of microbial exposure on the development of allergy and asthma. Therefore, the modern sanitized living standards of the developed world may contribute to disrupting the balance between our immune system and the microbiota that inhabit our environment^14^,^15^. This perspective is consistent with the finding that children at risk for developing asthma have altered intestinal microbiomes^16^, which have been attributed to the use of formula, probiotics, and/or antibiotics^17^. Therefore, means by which we are able to stimulate the immune system to overcome the dysfunction and dysbiosis caused by the reduced early life exposure to microbes may provide a new alternate avenue for managing the ever-increasing incidence of immune disorders such as asthma.

Different treatment strategies using bacterial products have shown clinical and *in vivo* efficacy at overcoming immune dysfunction in allergic disease, ranging from live bacteria that alter the microbiome, to specific pattern recognition receptor agonists^18^,^19^,^20^,^21^. In this study, we investigated whether using a novel therapeutic derived from a microbial species, *Klebsiella* (KB), that commonly causes lung infections would improve the immune dysfunction in an established asthma model.

The overall objective of this study was to test the use of KB in an asthma model consisting of two week exposure to intranasally administered house dust mite (HDM), which has previously been shown to develop a robust T_H_2 response, serum IgE increase and goblet cell hyperplasia^22^. Using a prophylactic intervention strategy, we hypothesized that KB would attenuate development of respiratory mucosal immune responses important in an allergic asthma phenotype, resulting in lower levels of markers of systemic allergic sensitization, attenuation of the T_H_2 responses in the lung, and reduced pathology. Here, we demonstrate that a prophylactic subcutaneous intervention with a novel therapeutic derived from *Klebsiella* attenuates the development of T_H_2 lung and systemic inflammation, and associated lung pathology, while not inhibiting allergic sensitization. Our data is consistent with the hypothesis that bacterial derived therapeutics are able to diminish immune dysfunction in allergic airway disease, providing a potential new treatment option to be further investigated in additional studies, including in models of established airway disease.

## Results

### HDM induces an increase in HDM-specific serum IgE levels, which is not attenuated by KB

**Figure 1:**
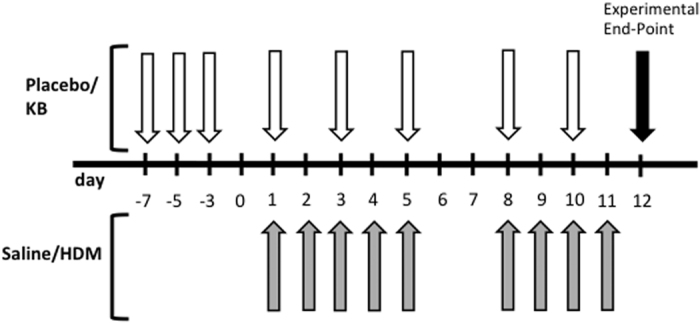
HDM sensitization and KB treatment protocol. Four groups of mice (n = 10) were exposed to either saline–placebo, house-dust-mite (HDM)–placebo, saline–KB compound, or HDM-KB compound. Grey arrows, intranasal HDM or saline exposure; white arrows, subcutaneous injection of placebo or KB. See methods for details.

In this study, we investigated the efficacy of a KB therapeutic at attenuating allergic sensitization and T_H_2-skewed lung inflammation in a HDM murine model. The model was a two-week intranasal HDM exposure model with euthanasia one day after the final exposure. Subcutaneous treatment with the KB therapeutic was given prophylactically for one week and continued throughout the HDM exposure phase (Fig. 1).

**Figure 2:**
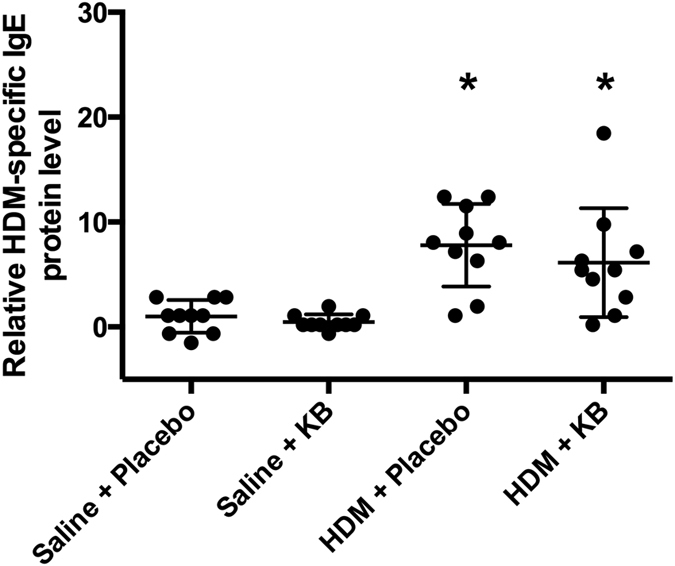
The impact of KB on HDM-specific serum IgE levels. Mice were exposed to saline or HDM, and treated with placebo or KB pre- and post exposure followed by challenges with HDM. Mouse HDM-specific serum IgE levels relative to placebo control 24 h after final exposure. Results are shown as mean ± SD (n = 10). *p < 0.05 compared to corresponding saline control.

As a measure of allergic sensitization, we measured HDM-specific IgE in the serum (Fig. 2). HDM exposure induced an increase in HDM-specific IgE when compared to saline treatment (p < 0.05). KB treatment did not significantly attenuate the HDM-specific IgE signal.

### KB attenuates lung inflammatory cell infiltrate following HDM exposure

**Figure 3:**
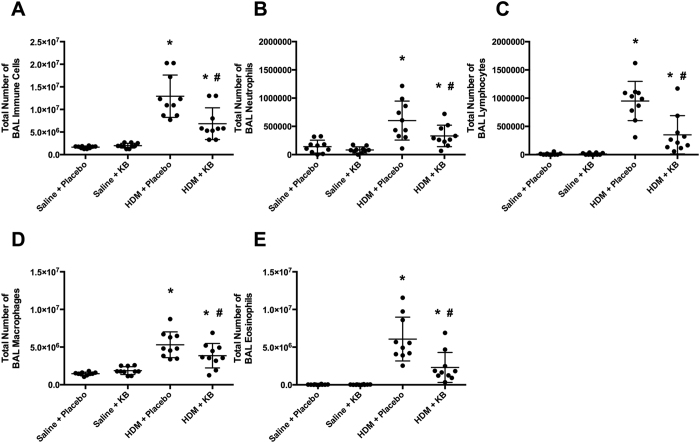
The impact of KB on lung inflammatory cell infiltrate following HDM exposure. Bronchoalveolar lavage (BAL) cell counts of mice exposed to saline or HDM, and treated with placebo or KB, with samples collected 24 h after final exposure. **(A)** Total cells. **(B)** Neutrophils. **(C)** Lymphocytes. **(D)** Macrophages. **(E)** Eosinophils. Results are shown as mean ± SD (n = 10). *p < 0.05 compared to corresponding saline control. ^#^p < 0.05 compared to corresponding placebo treated control.

As a measure of airway inflammation we performed bronchoalveolar lavages (BAL) and analysed the differential cell counts (Fig. 3). In placebo treated animals, HDM exposure induced an elevation in total BAL cell count (Fig. 3A, p < 0.05), which was attributed to an increase in neutrophils, lymphocytes, macrophages, and eosinophils (Fig. 3B–E, p < 0.05). KB decreased the total BAL cell count and the number of neutrophils, lymphocytes, macrophages and eosinophils in HDM exposed animals (p < 0.05). However, in HDM exposed animals treated with KB, the total BAL cell count and each cell type remained elevated relative to saline exposed mice treated with KB (p < 0.05). Within saline exposed mice, KB did not cause any significant change in the number of total cells, neutrophils, lymphocytes, macrophages or eosinophils in the BAL fluid.

### KB attenuates HDM-induced elevation of eotaxin and IL-5

**Figure 4:**
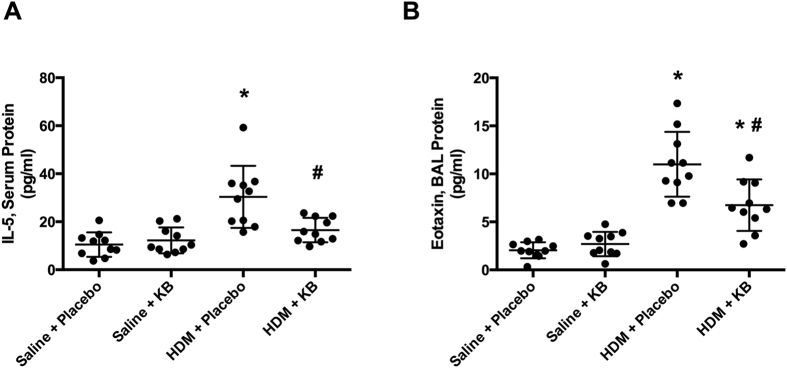
The impact of KB on HDM-induced elevation of eotaxin and IL-5. Mice were exposed to saline or HDM, and treated with placebo or KB. IL-5 and eotaxin levels were measured 24 h after final exposure. **(A)** Serum IL-5 levels. **(B)** BAL eotaxin levels. Results are shown as mean ± SD (n = 10). *p < 0.05 compared to corresponding saline control. ^#^p < 0.05 compared to corresponding placebo treated control.

Allergen-induced airway eosinophilia is influenced by systemic factors that regulate the differentiation of eosinophils in the bone marrow as well as chemotaxis to the lung^23^,^24^,^25^. IL-5 is a promoter of eosinopoiesis, promoting eosinophil maturation from the bone marrow and into the circulatory system^26^. Therefore, we next investigated if the suppression of HDM-induced airway eosinophilia was associated with changes in serum IL-5. HDM exposure induced an increase in serum IL-5 that was attenuated by KB (Fig. 4A). KB had no impact on serum IL-5 levels in saline exposed animals. Eotaxin is a eosinophil chemotactic factor that promotes eosinophil recruitment to the lungs^24^,^27^,^28^. In the lung, HDM exposure induced an increase in BAL eotaxin that was attenuated by treatment, although this remained elevated relative to saline controls (Fig. 4B). KB treatment had no impact on BAL eotaxin levels in saline exposed animals. Changes in other serum and BAL mediators can be found in Supplemental Tables 1 and 2.

### Increased expression of T_H_2 and T_H_1 genes in HDM exposed mice was not attenuated by KB

**Figure 5:**
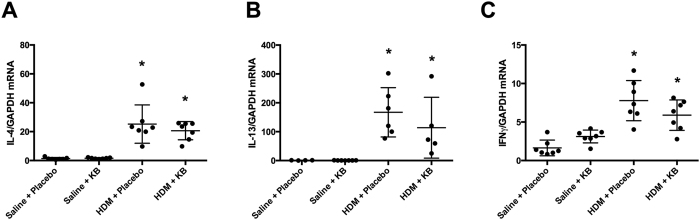
The impact of KB on T_H_2 and T_H_1 gene expression following HDM exposure. Mice were exposed to saline or HDM, and treated with placebo or KB. Lung T_H_2 associated **(A)** *Il-4* and **(B)** *Il-13*, and T_H_1-associated **(C)** *Ifng* gene expression levels relative to *Gapdh* were measured 24 h after final exposure. Results are shown as mean ± SD (n = 10). *p < 0.05 compared to corresponding saline control. ^#^p < 0.05 compared to corresponding placebo treated control.

Allergic airway inflammation and eosinophilia is associated with T_H_2-skewed immune profiles. Therefore, we next measured lung gene expression of select T_H_2 (IL-4 and IL-13) and T_H_1 (IFN-γ) genes in the lung tissue. HDM exposure induced an increase in IL-4 and IL-13 gene expression that was not attenuated by KB (Fig. 5A,B). KB also had no impact on baseline IL-4 and IL-13 gene expression. Similarly, IFN-γ gene expression was elevated in HDM-exposed groups and this was not attenuated KB (Fig. 4C).

### Increases in BAL T_H_2 cytokine protein levels following HDM exposure is attenuated by KB

**Figure 6:**
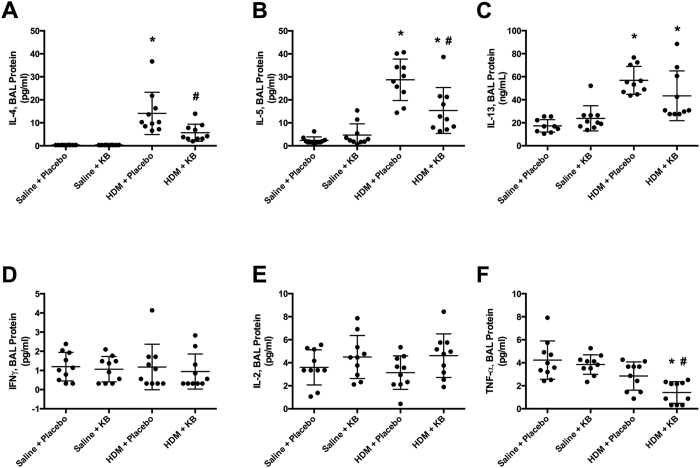
The impact of KB on BAL T_H_2 cytokine protein levels following HDM exposure. BAL cytokine levels in mice exposed to saline or HDM and treated with placebo or KB. Levels of T_H_2 cytokines **(A)** IL-4, **(B)** IL-5, and **(C)** IL-13 as well as T_H_1 cytokines **(D)** TNF-γ, **(E)** IL-2, **(F)** TNF-α, 24 h after final exposure. Results are shown as mean ± SD (n = 10). *p < 0.05 compared to corresponding saline control. ^#^p < 0.05 compared to corresponding placebo treated control.

As lung tissue gene expression may not accurately reflect protein production in the BAL, we performed multiplex and ELISA analyses that included T_H_2 and T_H_1 mediators of interest. HDM exposure induced an increase in the protein levels of the T_H_2 cytokines IL-4, IL-5 and IL-13 (Fig. 6A–C). KB attenuated HDM-induced IL-4 and IL-5, although IL-5 levels remained elevated relative to saline controls. Similarly, KB showed a trend for attenuation of HDM exposure induced IL-13 levels (p = 0.13). KB had no impact on BAL IL-4, IL-5, or IL-13 protein levels in saline exposed animals.

HDM exposure did not induce the T_H_1 cytokines, IFN-γ, IL-2, and TNF-α (Fig. 6D–F). KB did not impact IFN-γ or IL-2 in HDM-exposed animals, but did attenuate BAL TNF-α in these animals. KB treatment had no observable impact on BAL IFN-γ, IL-2, and TNF-α in saline exposed animals.

**Figure 7:**
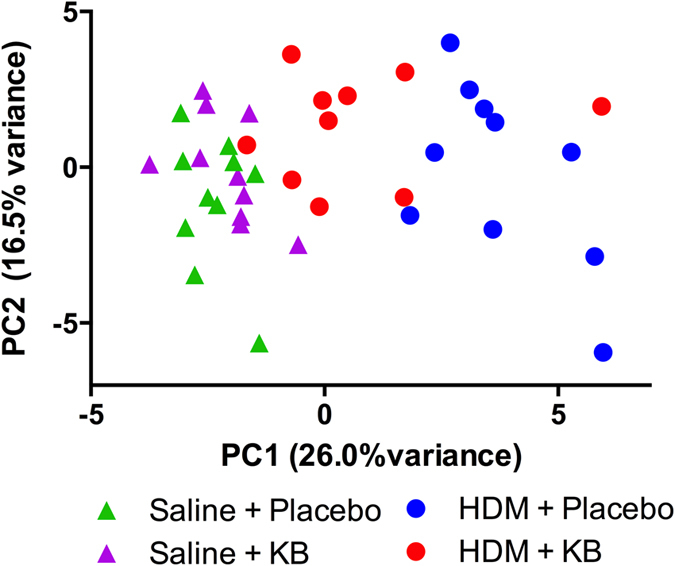
Principal component analysis (PCA) of cytokine mediators in BAL fluid. Each triangle on the graph and each circle represents one animal. Each colour represents one group. Green triangles: saline + placebo treated mice; Violet triangle: saline + KB; Blue circle: HDM + placebo; Red circle: HDM + KB. n = 10 per group.

To look at the overall BAL cytokine profile changes between groups, we did a principle component analysis (PCA) using all multiplex data and the IL-13 ELISA data. The different experimental groups clustered separately based on the 1^st^ principle component (PC1), containing 26% of the total variance (Fig. 7). Within the placebo treated mice, saline-exposed mice clustered separately from HDM exposed mice. KB treatment minimized the separation of HDM exposed mice from saline controls. Within the saline control group, KB and placebo treated mice were similar–as shown by their clustering together. As PC1 appeared to best differentiate the mice into different groups (explaining 26% of variance), we looked at the cytokines that had the greatest contribution to PC1. The top 5 cytokines that determined PC1 were IL-5 (7.3%), eotaxin (7.2%), LIF (Leukemia Inhibitory Factor; 7.2%), IL-4 (6.8%) and IL-13 (6.7%). Values of each mediator measured in BAL are shown in Supplemental Table 2.

### Increases in airway goblet cells following HDM exposure is attenuated by KB treatment

**Figure 8:**
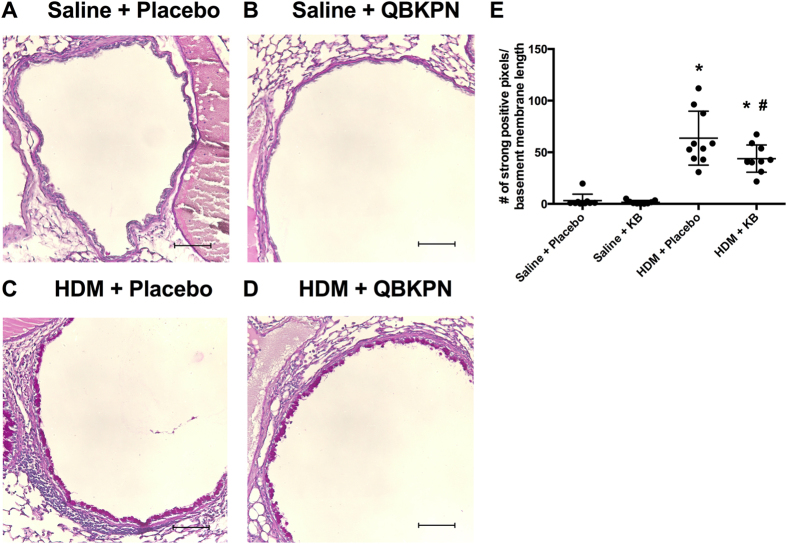
The impact of KB on HDM exposure induced goblet cell metaplasia. Photomicrographs of Periodic acid-Schiff stained lung tissue from mice exposed to saline or HDM, treated with placebo or KB **(A–D)**. Scale bar = 10 μm. **(E)** Quantification of mucus-containing goblet cells with the number of strong positive pixels normalized by basement membrane length (microns). Results are shown as mean ± SD (n = 10). *p < 0.05 compared to corresponding saline control. ^#^p < 0.05 compared to corresponding placebo treated control.

HDM exposure-induced T_H_2 skewed airway inflammation is associated with an increase in the mucus producing goblet cells, an important component of asthma pathology^29^,^30^. We therefore quantified the goblet cell number in the airways. HDM exposure induced an increase in the area of periodic acid-Schiff stained goblet cells. KB treatment reduced the number of goblet cells in HDM exposed animals, although this was still elevated relative to saline controls (Fig. 8). Treatment with KB had no impact on goblet cells in saline exposed animals.

## Discussion

Increasing evidence suggests that microbial exposures impact the development of the immune system and modulate the risk for developing allergic asthma^16^,^31^, which has led to the hypothesis that treatment of such disorders could involve agents that stimulate, rather than suppress, the immune system^21^. In our proof of concept study, we explored the ability of subcutaneous prophylactic treatment with a novel microbe-based compound, KB, to attenuate the establishment of allergic sensitization and lung inflammation in a mouse model of HDM exposure. Our results demonstrate that prophylactic treatment with KB attenuated markers of T_H_2-skewed allergic airway inflammation and goblet cell metaplasia, while not preventing the development of allergic sensitization. These results demonstrate efficacy and biological plausibility for future studies exploring how KB impacts established allergic airways disease with alternate dosing strategies aimed at reversing an established phenotype.

Allergic asthmatics demonstrate a T_H_2-cytokine skewed inflammatory profile with pronounced IL-4, IL-5, and IL-13 production in BAL and lung tissue^2^. T_H_2-skewed inflammatory responses contribute to the recruitment and activation of eosinophils, mast cells, and basophils to lung tissue. Activation of these cells can in turn impact mesenchymal cells including airway epithelial cells, smooth muscle cells, and fibroblasts, contributing to airway remodeling and airway hyperresponsiveness^1^. Selective targeting of T_H_2 cytokines to prevent allergic inflammation has been attempted with anti-IL-5, IL-4Ra and IL-13 antibodies and is currently used as add-on therapy options for patients that are poorly controlled with conventional corticosteroids^5^. HDM is a common model to trigger asthmatic responses and is known to induce IL-4, IL-5, IL-13 levels^30^,^32^. These cytokines were present in our study and BAL protein levels were attenuated to varying degrees by KB treatment. Principal component analysis completed on 31 BAL cytokines further showed the effect of KB on the HDM-induced T_H_2 response. In the principle component analysis, HDM exposed mice clustered independently from saline exposed mice, and KB treatment shifted the HDM exposed mice back towards the control mice. IL-4, IL-5, IL-13 and eotaxin were identified as the major contributing cytokines to this KB-induced shift. Collectively these results suggest a broad dampening of the T_H_2 response in the airways after treatment, which may have important clinical implications.

IgE levels have been therapeutically targeted in allergic asthma^33^. Allergen-specific IgEs are able to recognize epitopes on antigens, which have the potential to lead to the activation of eosinophils, mast cells, and basophils. Activation of these immune cells leads to the release of inflammatory mediators including the T_H_2 cytokines, IL-4, IL-5, and IL-13^1^. We demonstrated that HDM exposure induced a robust allergen-specific serum IgE response that was not affected by KB treatment. This finding suggests that the *Klebsiella* derived therapeutic may not be effective in preventing the development of allergic sensitization and allergen-specific IgE, while still being efficacious for controlling lung inflammation. Our mouse model observations showing a disconnect between systemic allergic sensitization and allergen-induced lung inflammation parallel a clinical scenario where atopic non-asthmatic individuals show reduced lung inflammation relative to atopic asthmatics^34^. Interestingly, our data suggests that in this allergen model, the production and regulation of T_H_2 cytokines are uncoupled from IgE levels. Furthermore, our data suggests that allergic outcomes of clinical importance (e.g. BAL eosinophils) may not be directly related to allergen-specific IgE levels in the serum.

Eosinophils are recruited to the lungs during allergic asthma and mouse models of HDM exposure^30^. Eosinophils express the high affinity FceR that binds allergen specific IgE, which is a pre-requisite for activation and release of inflammatory mediators. Eosinophil levels are impacted by systemic factors that activate the bone marrow to produce eosinophils, and chemokines to recruit them to the lung^23^,^24^,^25^. Eotaxin can be released from airway epithelial cells, smooth muscle cells, and fibroblasts to recruit eosinophils following an allergen exposure^27^. Eotaxin is a potent chemokine for eosinophils and has served as a predictor of asthma onset and disease severity^27^. In contrast to eotaxin, IL-5 is a potent survival factor for eosinophils and promotes the maturation of progenitors leaving the bone marrow^35^,^36^. Clinical targeting of IL-5 for asthma therapy implicates the importance of this mediator in certain populations of asthmatics^10^. Therefore, for this treatment strategy to be efficacious at attenuating the downstream consequences of eosinophil activation by FceR cross-linking, an effective alternate strategy would be to reduce the total number of airway eosinophils or the ability for them to be recruited to the lung. Consistent with this biological concept, we demonstrate that HDM exposure induced an elevation in serum IL-5 and BAL eotaxin that are both attenuated by KB treatment. Our results indicate that a treatment with a biologic is capable of simultaneously targeting two of the key molecular components that promote airway eosinophilia, in a process that is independent of the regulation of allergen specific IgE.

Microbes and microbial products can stimulate the immune system, shifting the immune profile to have positive benefits on asthma phenotypes. Live, orally delivered, bacteria have shown promise in preclinical studies at reducing T_H_2 responses. For example, similar to KB, oral exposure to live *Lactobacillus reuteri* bacteria can attenuate IL-5, IL-13 and eosinophilia in a mouse model of allergic airway inflammation, which was accompanied by a decrease in airway hyperresponsiveness^19^. Interestingly, oral administration of heat-killed *Lactobacillus* also reduced IL-5, TNFα and MCP-1 BAL levels, although this did not alter airway total cells or airway hyperresponsiveness^19^. Specific microbial products can also have potential benefits on asthma phenotypes. For example, lipopolysaccharide (LPS), which is found on gram negative bacteria including *Klebsiella,* have shown protective effects and can decrease the T_H_2 response when delivered intranasally and intravenously^37^,^38^,^39^. Other specific bacterial derived products that are agonists for pattern recognition receptors have the potential to decrease the T_H_2 response including macrophage-activating lipopeptide-2 (MALP-2) from *Mycoplasma fermentans*^40^ and Lipoprotein 1 (Opr1) isolated from *Pseudomonas aeruginosa*^41^. Mechanistically, these approaches are thought to prime dendritic cells and cause release of IFN-γ, ultimately leading to a decreased T_H_2 response^40^,^41^. Overall, this supports the approach of using bacterial products to alter the lung immune response. Future studies will be aimed at identifying the specific pathway and molecules through which the KB compound provides efficacy.

Mouse models of allergen exposure have been important for studying allergic sensitization, lung inflammation and associated lung pathologies^42^,^43^. The HDM exposure models in mice recapitulate the allergic sensitization, lung inflammation, pathology, and airway responsiveness that are observed in human asthmatics^44^. In this study, we showed that prophylactic treatment with KB in a two week model of intranasal HDM exposure effectively attenuates total BAL cells counts, eosinophilia, BAL T_H_2 cytokines, markers of eosinophil development and migration, and lung pathology. In light of our results, future studies examining the efficacy of KB in T_H_17 skewed mouse models of steroid resistant human asthma^45^ or T_H_2-low endotypes^2^,^6^ are warranted, which should include more refined analysis of lung and blood immune cell phenotypes (e.g. T regulatory cells)^46^ and activity states to extend understanding of how KB modulates immune responses important in allergic airways disease.

In conclusion, these data show that the a prophylactic microbe-derived treatment, KB, is efficacious at attenuating HDM-induced T_H_2 skewed allergic airway inflammation, airway eosinophilia, and mucus content in goblet cells, independent of modulating allergen-specific IgE levels. Our study demonstrates proof of concept that this is a relevant novel treatment strategy to modulate inflammatory processes in asthma and sets the foundation for testing efficacy for reversal of established allergic airways disease. KB may promote a switch in the immune system, providing a novel approach to treat patients with asthma or potentially serving as an early-in-life immune modulating approach to the prevention of asthma for high-risk individuals.

## Methods

### Animals

Female mice (BALB/c) age 6–8 weeks old were purchased from Jackson Laboratory (Bar Harbor, ME). 10 mice per group were used. Mice were housed in environmentally controlled specific pathogen free conditions with a 12:12 hour light/dark cycle for the duration of the study. All protocols were reviewed and approved by the Animal Research Ethics Board of University of British Columbia (Vancouver, BC, Canada) and all studies were carried out in accordance with the Canadian Council for Animal Care guidelines. No deviations from the guidelines were performed.

### Allergen exposure protocol

Mice were exposed to saline (35 μL) or house dust mite (HDM, *Dermatophagoides pteronyssimus*, Greer Laboratories, Lenoir, NC), intranasally, 25 μg in 35 μL of saline, under isoflurane anesthesia^22^. The HDM was product number XPB70D3A25, lot number 231897 with an endotoxin content of 78.72 endotoxin units/mg with an expiration date of 2023. HDM or saline nasal exposure was done for 5 consecutive days in week 1 and 4 consecutive days in week 2 (experimental days: 1–5; 8–11, Fig. 1). Mice were euthanized 24 hours after the last exposure.

### *Klebsiella* intervention strategy

KB was an immunogenic product derived from a propriety *Klebsiella* strain originally isolated from a sputum sample of a patient with acute *Klebsiella* pneumonia (Qu Biologics Inc., Vancouver, BC). The product comes suspended in physiological saline containing 0.4% phenol as a preservative for a final OD_600_ of 5.0. Placebo was physiological saline containing 0.4% phenol. KB or placebo was prophylactically given on day −7, −5, −3 of the experiment, and treatment was continued on experimental days 1, 3, 5, 8, 10. 30 μL of placebo or KB was injected at alternating sites subcutaneously in the lower right and left quadrant of the abdomen and the upper right and left quadrant of the chest, as suggested in the product protocol.

### Blood collection, BAL, and cytospin analysis for BAL cell differentials

Blood and BAL were collected and analysed as described previously^29^. BAL cell differential counts were performed by examining cytospins according to cell morphology and Wright-Giemsa staining. A total of 100 cells per mouse were differentiated by a blinded observer.

### Quantification of HDM-specific immunoglobulins by ELISA

HDM-specific IgE expression was analysed using a modified protocol^47^. In brief, HDM was coated onto 96-well plates (2.5 ug/well) and incubated overnight at 4 °C. After blocking with 5% FBS in PBS, undiluted serum was added and incubated overnight at 4 °C. After washing, biotin anti-mouse IgE (BD Bioscience-San Jose, CA, USA) was added and incubated at 37 °C for one hour. Streptavidin-HRP/TMB substrate was used to visualize levels and absorbance was recorded at 450 nm. Data is presented as relative to the placebo treated saline exposed group.

### Gene expression

Right lung tissue was lysed by homogenizing with a TissueLyser LT (Qiagen–Toronto, Ontario, Canada) and RNA isolation performed using a PureLink RNA Mini Kit (Life Technologies-Carlsbad, CA, USA). iScript cDNA Synthesis Kit was used for cDNA synthesis (Biorad). Gene expression was done by quantitative RT-PCR on a StepOnePLus RT-PCR machine (Applied Biosystems-Foster City, CA, USA) using TaqMan Fast Advanced Master Mix (Applied Biosystems) with Taqman probes for IL-4 (Mn00445259_m1), IL-13 (Mn00434204_m1) and IFN-γ (Mn01168134_m1).

### Cytokine and chemokine analysis of BAL and serum samples

We quantified 31 cytokine/chemokine/growth factor biomarkers simultaneously using a Milliplex Mouse Cytokine/Chemokine kit (Millipore, St. Charles, MO, USA) according to the manufacturers protocol. The multiplex was performed by Eve Technologies (Eve Technologies Corp, Calgary, AB, Canada) using the Bio-Plex 200 system (Bio-Rad Laboratories, Inc., Hercules, CA, USA). The 31-plex consisted of eotaxin, G-CSF, GM-CSF, IFNγ, IL-1α, IL-1β, IL-2, IL-3, IL-4, IL-5, IL-6, IL-7, IL-9, IL-10, IL-12 (p40), IL-12 (p70), IL-13, IL-15, IL-17, IP-10, KC, LIF, MCP-1, M-CSF, MIG, MIP-1α, MIP-1β, MIP-2, RANTES, TNFα, and VEGF. The assay sensitivities of these markers range from 0.1–33.3 pg/mL. As IL-13 levels in the multiplex were mainly below detection, IL-13 protein levels were measured in the BAL fluid by an ELISA (eBioscience San Diego, CA, USA).

### Histology

Lungs were dissected and inflated with 5 mL of 10% formalin as described previously^29^. Tissues were embedded with paraffin and sectioned at 3 μm. Sections were stained with Periodic acid-Schiff to quantify mucus-containing goblet cells. Stained sections were scanned at 60X magnification using an Aperio Slidescanner (Vista, CA), version 11.1.2.760. Positively stained pixels were identified by colour segmentation in a cross-sectional manner in the lung airway using Aperio Image Scope software to express the number of strong positive pixels (Periodic acid-Schiff) normalized to basement membrane length (μM).

### Data Analysis

Data were analysed using GraphPad Prism and are expressed as mean ± SD. Multi-group comparisons were made by one-way ANOVA followed by Sidak post-hoc test. Four experimental group combinations of interest were compared; Saline-placebo vs. Saline- *Klebsiella*, Saline-placebo vs. HDM-placebo, Saline-*Klebsiella* vs. HDM- *Klebsiella*, HDM-placebo vs. HDM- *Klebsiella*. Differences were considered to be statistically significant when p < 0.05. For the purpose of statistical analysis, any value that was below the lowest value of the standard was recorded as half the lowest value of the standard. Principal component analysis (PCA) was performed for the BAL multiplex data, shown in Supplemental Table 1, plus the IL-13 ELISA. PCA was completed in R (version 3.2.4) using the prcomp command. (R Core Team (2016) R: A language and environment for statistical computing. R Foundation for Statistical Computing, Vienna, Austria. URL https://www.R-project.org/).

## Additional Information

**How to cite this article**: Bazett, M. *et al*. A novel *microbe-based* treatment that attenuates the inflammatory profile in a mouse model of allergic airway disease. *Sci. Rep.* **6**, 35338; doi: 10.1038/srep35338 (2016).

## Supplementary Material

Supplementary Table 1

Supplementary Table 2
